# The impact of fillers on lineup performance

**DOI:** 10.1186/s41235-017-0084-1

**Published:** 2017-11-22

**Authors:** Stacy A. Wetmore, Ryan M. McAdoo, Scott D. Gronlund, Jeffrey S. Neuschatz

**Affiliations:** 1 Butler University, Indianapolis, IN USA; 2 University of Oklahoma, Norman, OK USA; 3 The University of Alabama in Huntsville, Huntsville, AL USA

**Keywords:** Filler siphoning, WITNESS model, Eyewitness identification, Showups, Simultaneous and sequential lineups

## Abstract

Filler siphoning theory posits that the presence of fillers (known innocents) in a lineup protects an innocent suspect from being chosen by siphoning choices away from that innocent suspect. This mechanism has been proposed as an explanation for why simultaneous lineups (viewing all lineup members at once) induces better performance than showups (one-person identification procedures). We implemented filler siphoning in a computational model (WITNESS, Clark, Applied Cognitive Psychology 17:629–654, 2003), and explored the impact of the number of fillers (lineup size) and filler quality on simultaneous and sequential lineups (viewing lineups members in sequence), and compared both to showups. In limited situations, we found that filler siphoning can produce a simultaneous lineup performance advantage, but one that is insufficient in magnitude to explain empirical data. However, the magnitude of the empirical simultaneous lineup advantage can be approximated once criterial variability is added to the model. But this modification works by negatively impacting showups rather than promoting more filler siphoning. In sequential lineups, fillers were found to harm performance. Filler siphoning fails to clarify the relationship between simultaneous lineups and sequential lineups or showups. By incorporating constructs like filler siphoning and criterial variability into a computational model, and trying to approximate empirical data, we can sort through explanations of eyewitness decision-making, a prerequisite for policy recommendations.

**Electronic supplementary material:**

The online version of this article (doi:10.1186/s41235-017-0084-1) contains supplementary material, which is available to authorized users.

## Significance

We apply a formal model to the recently proposed filler siphoning explanation for why performance from simultaneous lineups is superior to showups. We show that, although fillers can produce a simultaneous lineup performance advantage, the magnitude of that advantage is insufficient to fit empirical data. Although the addition of criterial variability allows performance differences to be approximated, it is not because of a positive contribution from filler siphoning, but rather the greater adverse impact of criterial variability on showups. Theoretical understanding rooted in formal models is a prerequisite for policy recommendations in applied domains.

## Background

Researchers have begun using receiver operating characteristic (ROC) analysis to assess the degree to which eyewitnesses distinguish innocent from guilty suspects when tested with simultaneous lineups, sequential lineups, and showups (e.g., Gronlund et al., 2012; Mickes, Flowe, & Wixted, 2012; Neuschatz et al., 2016; Wetmore et al., 2015). One result emanating from these studies is that simultaneous lineup performance is superior to showups (for a review, see Clark, 2012), and Wetmore et al. (Gronlund et al., 2012, Neuschatz et al., 2016; Wetmore et al., 2015) argued that simultaneous lineup performance was superior because lineups afford better discriminability than showups (see Wixted & Mickes, 2014). However, this interpretation caused some alarm, which was spotlighted in a series of papers (Wells, Smalarz, & Smith, 2015; Wells, Smith, & Smalarz, 2015; but see Wixted & Mickes, 2015a, 2015b). The crux of this debate focused on whether simultaneous lineups are superior to showups because of increased discriminability (increased ability to distinguish the guilty and innocent suspects) or filler siphoning (defined below).

Wells, Smalarz, et al. (2015) proposed two arguments for why discriminability was an inappropriate interpretation of the lineup advantage. The first was that ROC analysis imposes an inappropriate response structure by forcing a 3 × 2 response structure into a 2 × 2 response structure. The 2 × 2 structure they referred to was the classic signal-detection structure (2 stimulus types: noise vs. noise + target × 2 responses: positive or negative). That is, on every trial with a 2 × 2 structure, a decision must be made regarding whether a previously shown stimulus was studied or not. If the stimulus was studied before and a participant responds positively, it is a hit; if a participant responds negatively, it is a miss. Conversely, if the stimulus was not studied before but the participant responds positively, then this is a false alarm, and if the participant responds negatively, it is a correct rejection. Lineups, however, correspond to a 3 × 2 structure due to the presence of known-innocent fillers. Participants are presented with a target-present (includes the guilty suspect) or a target-absent (the guilty suspect is replaced by an innocent suspect) lineup, and can make a positive selection of a suspect, a positive selection of a filler, or a negative response rejecting the lineup. Because ROC analysis focuses on suspect identifications (IDs) (correct IDs of the guilty suspect from target-present lineups and false IDs of a designated innocent suspect^1^ from a target-absent lineup), Wells et al. argued that the positive selection of a filler was treated as a rejection, which made ROC analysis misleading (see Rotello & Chen, 2016, for a counterargument). This ignoring of fillers is central, given the explanation that Wells et al. favor.

The second argument that Wells et al. proposed was that filler siphoning was responsible for the superiority of lineups over showups. That is, filler siphoning theory posits that the fillers in a target-absent lineup protect an innocent suspect from being chosen by siphoning or shifting choices from the innocent suspect to the fillers. Of course, fillers also can siphon choices from a guilty suspect, but it has been argued that more choices will be siphoned from an innocent than from a guilty suspect because a guilty suspect should match memory better (termed differential filler siphoning, Smith, Wells, Lindsay, & Penrod, 2017). However, the absence of fillers in a showup provides no such protection for an innocent suspect. Because the choice of a filler could be construed as a false positive, Wells et al. argued that it signaled a difficulty in discriminability that was ignored by ROC analysis as applied to lineups. Consequently, they argued that what was being measured by ROC analysis cannot be discriminability.^2^ In sum, although Wells et al. agreed with Wetmore et al.’s empirical conclusion – simultaneous lineups are superior to showups – they did not agree that enhanced discriminability was the reason. Rather, differential filler siphoning was the reason that lineups result in superior performance. It is this second argument that we explore.

It is challenging to extract definitive predictions from a verbally specified explanation, such as filler siphoning, because the lack of formalism obscures its workings (see Bjork, 1973; Lewandowsky, 1993). Explanations that are not formally specified are too flexible, which makes them difficult to evaluate. For example, is one filler enough to demonstrate filler siphoning? Does one filler provide as much protection for an innocent suspect as 3, 6, 12 or 50 fillers? Or should there be a concomitant increase in siphoning (fewer false identifications) as lineup size increases, provided all fillers are viable options? A formally specified model, on the other hand, forces a theoretician to be explicit about a model’s assumptions. This makes transparent the reasons for its predictions, as well as providing a check on reasoning biases (Hintzman, 1991).

Many examples exist of verbal explanations leading researchers astray. To take just one example, do we summarize our knowledge about a category (e.g., birds) by storing in memory a summary prototype (a depiction of the average bird), or do we instead store all the category exemplars that we experience? Posner and Keele (1970) showed that participants responded to a tested prototype more strongly than to any experienced exemplar, even though the prototype had never been experienced. This was thought to provide strong evidence for the psychological reality of categorization decisions being based on the representation of a single prototype. But Hintzman (1986) took a formally specified exemplar model and reproduced the same performance advantage for the test of a prototype. The exemplar model accomplished this because it made decisions by matching a test item to everything in memory. Although a tested prototype exactly matched nothing in memory, as the “average” stimulus, it closely matched all the stored exemplars, resulting in a strong response from memory. In sum, a formal model was used to demonstrate that data thought to definitively require the storage of prototypes, did nothing of the sort.

It is for reasons like this that it is necessary to explore filler siphoning theory using a formally specified model. We employ the WITNESS model to achieve this goal (see also Clare & Lewandowsky, 2004; Clark, 2003; Clark, Erickson, & Breneman, 2011; Fife, Perry, & Gronlund, 2014; Goodsell, Gronlund, & Carlson, 2010). The WITNESS model is the first formal model designed for eyewitness decision-making; its parameters tie directly to key components of the eyewitness task. But because WITNESS shares many characteristics with signal detection applications to recognition memory (Banks, 1970), it is likely that what we can learn about filler siphoning using WITNESS is similar to what we would learn had we modified a different computational model of memory (for reviews of these types of models see Clark & Gronlund, 1996; Malmberg, 2008; Rotello & Chen, 2016).

## Methods

### WITNESS model

The WITNESS model (Clark, 2003) is a direct-access matching model designed to explore and understand the variables that affect eyewitness ID. The details of the WITNESS model are beyond the scope of this article and the interested reader is directed to Clark (2003). Instead, we highlight the aspects of the model necessary for our analysis.^3^

The WITNESS model simulates an eyewitness ID procedure in two stages: construction and decision. In the construction phase, the model “builds” a number of elements: a perpetrator, the memory that an eyewitness retains of the perpetrator, the other members of the lineup (fillers), and for the target-absent lineups, an innocent suspect. Parameters of the model govern how well the perpetrator is encoded into memory, and the degree to which the innocent suspect and fillers resemble the perpetrator. The decision stage specifies how these various elements are assessed and how a decision is made from the ID procedure. How these elements are constructed, and how the parameters determine ID rates, are described next.

In the model, a perpetrator (PERP) is specified as a vector of 100 random values between − 1 and + 1. Each element in the vector symbolizes a characteristic of the perpetrator (e.g., size of the nose, shape of the mouth, hair color, hair texture). Once PERP is created, it is encoded into memory (MEM). The quality of encoding is governed by the parameter *a*, whereby a feature is correctly encoded into MEM (derived from PERP) with probability *a*, and noise, a new random value between − 1 and + 1, is encoded with probability (1 – *a*). For example, if *a* = .25, 25% of MEM will match the PERP and 75% will consist of random values. The creation of the innocent suspect and the fillers follows a similar logic whereby the parameters *SSP* (Similarity of the innocent Suspect to the PERP) and *SSF* (Similarity of the Suspect to the Fillers) govern the probability of the innocent suspect and filler features matching the PERP, respectively. For example, if *SSP* = .7, then 70% of the features match between the perpetrator and the innocent suspect. The closer *SSP* (or *SSF*) is to 1.0, the more the innocent suspect (or the fillers) resembles the perpetrator. When *SSP* = *SSF*, then the innocent suspect and fillers resemble the perpetrator to the same degree (i.e., a fair lineup). In addition, the type of ID procedure, either a simultaneous or sequential lineup or showup, can be specified in the model. Lineups are modeled by compiling the proper number of vectors (typically 6), which includes the guilty suspect (PERP) with five fillers in target-present lineups, or the innocent suspect with five fillers in target-absent lineups. A showup (a one-person lineup) is created by including only the PERP or the innocent suspect.

Once the vectors have been specified, the model makes a decision. For a simultaneous lineup, the model begins by computing the degree to which each lineup member matches memory for the perpetrator. These match values are computed by taking the dot product of each lineup member vector and MEM. A decision is made from the simultaneous lineup by comparing the highest match value (i.e., the best) to a decision criterion, the value of which is governed by the parameter *csim*. If the best-match value fails to exceed *csim*, in either target-present or target-absent conditions, a rejection is recorded. If the best-match value exceeds *csim*, the model selects that lineup member. If the best match is the guilty suspect in a target-present lineup, then the selection is a correct ID, if not it is a filler ID. If the best match is the innocent suspect in a target-absent lineup, then the selection is a false ID, otherwise it is a filler ID. This is an implementation of an absolute decision rule.^4^ Showup decisions are made similarly. A match value is calculated and compared to a decision criterion, *csu*.^5^ If the match value falls above *csu*, a correct ID (in target-present) or false ID (in target-absent) is recorded. If the match value falls below *csu*, a rejection is recorded.

For a sequential lineup, the lineup members are matched to memory in sequence, and in the version we implemented, evaluation stopped as soon as a match value exceeded the decision criterion *cseq* (see also Goodsell et al., 2010). If the end of the lineup was reached without any match value exceeding *cseq*, a reject decision was recorded. Position of the suspect was randomized for each simulation.

The construction and decision phases are repeated 10,000 times to simulate 10,000 participants making an eyewitness decision. The output of the model provides estimates of the proportions of correct IDs, false IDs, filler IDs, and rejections for a given set of parameter values. Along with ID rates, the WITNESS model can also be used to construct ROC curves. The construction of empirical ROC curves in the eyewitness domain relies on participant confidence ratings. For instance, after making an ID, a witness is asked how confident he or she is in the decision on a scale of (for example) 1 to 10:1 indicates that the witness is not confident at all, and 10 indicates the witness is highly confident in the decision. In the model, confidence ratings are apportioned by dividing the match strength dimension (the range of values from the decision criterion to the best possible match value) into the number of bins that correspond to the confidence scale (e.g., 10 bins for a confidence scale from 1 to 10). When a selection is made from a lineup or showup, the selection’s match value is compared to the various confidence bins, and a tally is added to the bin in which the value falls. For instance, if the match value for the ID of a suspect was 2.0, the model will tally a confidence response of 8 if confidence bin 8 extends from 1.9 to 2.1.

The present research explores the impact of fillers throughout WITNESS’ theory space. A theory space exploration varies the parameters over a wide range to see what the model can predict. According to filler siphoning theory, the presence of fillers is sufficient to produce a lineup advantage, which means that a lineup advantage should be apparent throughout the theory space. However, we expected to see that the magnitude of the lineup advantage would increase as the number of fillers (lineup size) increases, and as the competitiveness of the fillers (filler quality) increases. This is because adding fillers that are close competitors to a guilty suspect (higher values of *SSF*), should induce more siphoning, and thereby provide more protection for innocent suspects (thereby decreasing the number of false IDs).

The theory space exploration will proceed as follows: We begin by focusing on the same comparison as Wells et al. (Smith et al., 2017; Wells, Smalarz, et al., 2015), simultaneous lineups vs. showups, and then we move to an assessment of sequential lineups, which Wells et al. never considered. Although Smith et al. (2017) suggested that criterial variability is necessary for filler siphoning to emerge, the original instantiation did not incorporate variability (Wells, Smalarz, et al., 2015). Therefore, we first evaluate the original proposal, and fit empirical data, before exploring the impact of additional mechanisms.

### Theory space exploration

The first set of simulations varied the competitiveness of the fillers by varying the value of *SSF* in fair simultaneous lineups, and setting *SSF* = *SSP* (the innocent suspect does not stand out from the fillers). In this case, the innocent suspect and fillers are of equal similarity, which should result in the greatest amount of siphoning (protection for the innocent suspect). The second set of simulations also varied the competitiveness of the fillers, but in this case the simultaneous lineups were biased against the innocent suspect (*SSP* > *SSF*, the innocent suspect more closely resembled the perpetrator than did the fillers).

To begin, parameter values were selected based on values that have been used to fit empirical data (Goodsell et al., 2010). Goodsell et al. fit the WITNESS model to 10 sets of data and found that the encoding parameter *a* ranged from .11 to .33. We report results using a value of *a* = .3 for our simulations (the simulations were repeated with *a* = .2 and *a* = .5 and the pattern of results did not differ from those reported). The values for *SSP* and *SSF* varied over a wide range (.25 to .92, and .15 to .51, respectively), reflecting the wide differences across experiments in the degree of match of innocent suspects and fillers to a perpetrator. The *SSP* and *SSF* values used in the current simulations were varied over comparable ranges (.3 to .75). There were no indications amongst the many simulations we conducted that the general patterns we report next were unique to the particular parameter values used. Moreover, to reiterate, there is nothing special about using WITNESS to perform these explorations – alternative signal detection and matching models should behave similarly.

## Results

### Fair simultaneous lineups

For the first set of simulations, the match of the innocent suspect was set equal to the fillers to approximate a fair lineup (*SSP* = *SSF*). Filler siphoning theory predicts that when lineups are compared to the showups in this manner, lineups should exhibit an advantage. Additionally, we reasoned that as lineup size increases, more siphoning should occur (lineups should exhibit increasingly better performance than showups) because there are more viable options (more opportunities to siphon).

**Fig. 1:**
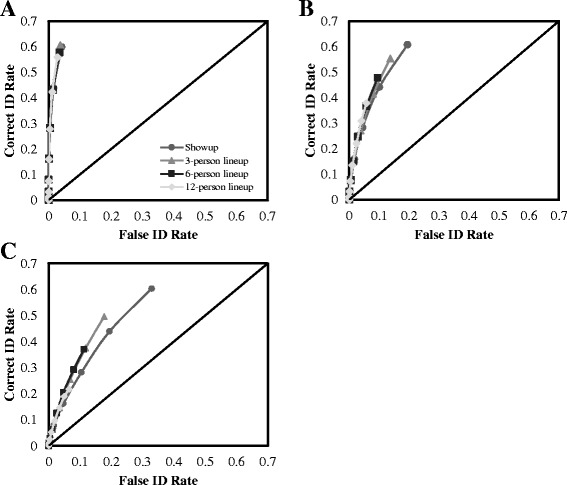
Receiver operating characteristic (ROC) curves comparing showups, 3-, 6-, and 12-person fair lineups in which *SSP* = *SSF*. **a** *SSP* = *SSF* = .3, **b** *SSP* = *SSF* = .6, **c** *SSP* = *SSF* = .75. Encoding fixed at *a* = .3, and *csim* = *csu* = .09. The solid diagonal line indicates chance performance (guilty and innocent suspects chosen at the same rates). *SSP* Similarity of the innocent Suspect to the PERP (perpetrator), *SSF* Similarity of the Suspect to the Fillers

A 4 (lineup size: 1, 3, 6, and 12) × 3 (suspect/filler similarity (*SSP* = *SSF*: .3, .6, and .75) factorial was conducted. Figure 1 shows the resulting ROCs for showups (lineup size = 1), and 3, 6, and 12-person lineups, for *SSP* = *SSF* = .3 (panel A), where the innocent suspect and fillers share 30% of features with the PERP, *SSP* = *SSF* = .6 (panel B), where the innocent suspect and fillers share 60% of features with the PERP, and *SSP* = *SSF* = .75 (panel C), where the innocent suspect and fillers share 75% of features with the PERP. All guilty/innocent suspect, filler, and rejection rates for all simulations are available in Additional file 1.

In panel A, there is no separation between the ROC curves for the showup, 3-, 6-, or 12-person lineups. This replicates the findings of Rotello and Chen (2016, Fig. 1), although they implemented an equal variance signal detection model. However, the innocent suspect is chosen so seldom that there is little opportunity for siphoning to further limit that choosing. Panels B and C increase the degree to which the innocent suspect matches the perpetrator, thereby increasing the rate at which the innocent suspect is chosen. Panel B shows that the ROC curves separate slightly over the low confidence range of responses (the right-hand end of the curves). Panel C shows a separation between the showup and lineups; however, there is no separation amongst the different lineup sizes. These latter results differ somewhat from Rotello and Chen (2016, p. 4), who reported, in reference to their Fig. 1, that “Other values of true *d*′ yielded similar results” (i.e., no differences).

In sum, as the degree to which the innocent suspect and the fillers resemble the perpetrator is increased, some separation can be seen in the ROC curves. However, contrary to filler siphoning theory, the impact of increasing the number of fillers beyond three did not affect discriminability (the distance of the ROC above the chance diagonal); rather, it truncated the ROC curves (because fillers are being selected instead of innocent or guilty suspects).

### Biased simultaneous lineups

**Fig. 2:**
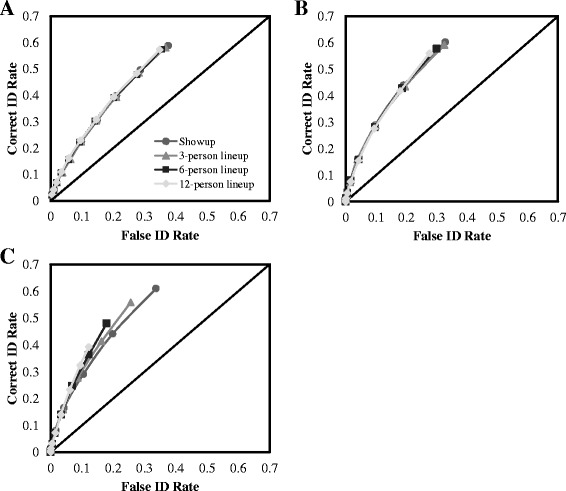
Receiver operating characteristic (ROC) curves comparing showups, 3-, 6-, and 12-person biased lineups *SSP* = .75, making the innocent suspect a better match to the PERP than the fillers. **a** *SSF* = 0.0, **b** *SSF* = .3, **c** *SSF* = .6. Encoding constant at *a* = .3, and *csim* = *csu* = .09. The solid diagonal line indicates chance performance. *SSP* Similarity of the innocent Suspect to the PERP, *SSF* Similarity of the Suspect to the Fillers, *PERP* perpetrator

The second set of simulations also varied the competitiveness of fillers, but in this case the lineups were biased (*SSP* > *SSF*, the innocent suspect more closely resembled the perpetrator than did the fillers). The innocent suspect was set to be *SSP* = .75 and held constant while varying *SSF*. Across panels in Fig. 2, the innocent suspect remains a very good match to the perpetrator, but the fillers become increasingly competitive as *SSF* is increased, which should affect the degree of protection afforded to the innocent suspect. Once again, we varied the similarity of the fillers in a 4 (lineup size: 1, 3, 6, and 12) × 3 (filler similarity (*SSF*): .0, .3, and .6) factorial. The three panels in Fig. 2 show the ROCs for the various lineup sizes with *SSF* = 0 (panel A), *SSF* = .3 (panel B), and *SSF* = .6 (panel C).

When fillers are poor or fair matches to the perpetrator (Fig. 2, panels a and b), the number of fillers has no effect. But as the fillers become stronger competitors (panel c), we see evidence consistent with the claims of filler siphoning theory (note that Fig. 2, panel c is similar in parameter values to Fig. 1, panel b). However, the claim by Wells, Smalarz, et al. (2015, p. 315) that fillers should be more protective in fair than in biased lineups was not apparent. Instead, fillers have different consequences for fair (Fig. 1) and biased (Fig. 2) lineups. When the innocent suspect is just another filler (*SSP* = *SSF*, as in Fig. 1), increasing lineup size does not affect the discriminability of the different lineup sizes, it only serves to truncate the ROCs. But when the innocent suspect is a better match to memory than the fillers (biased lineups), increasing the number of fillers did increase the level of the ROC as lineup size increases (although it also truncates it).

### Fair sequential lineups

Wells et al. (Smith et al., 2017; Wells, Smalarz, et al., 2015; Wells, Smith, et al., 2015) never addressed sequential lineups in their articles about filler siphoning, but sequential lineups, of course, also have fillers. Recommendations have been made for police departments to conduct sequential rather than simultaneous lineups to enhance performance (e.g., Well et al., 1998), although questions have arisen regarding this recommendation in recent years (for a review, see Gronlund, Mickes, Wixted, & Clark, 2015). Fillers should operate the same way in a sequential lineup as they do in a simultaneous lineup; consequently, filler siphoning theory predicts that as more and better fillers are added to a sequential lineup, performance should increase relative to a showup.

**Fig. 3:**
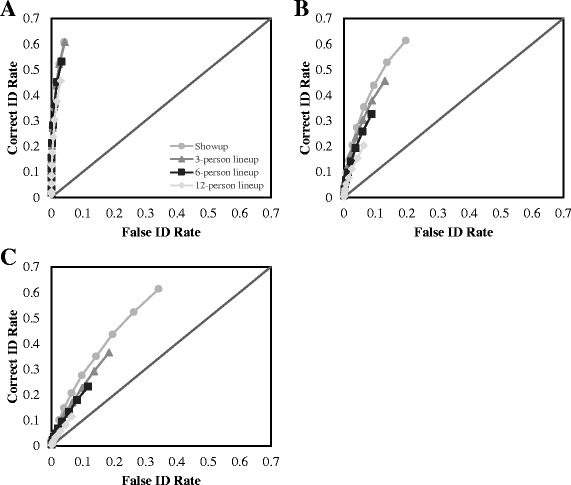
Receiver operating characteristic (ROC) curves comparing showups, 3-, 6-, and 12-person fair sequential lineups. **a** *SSP* = *SSF* = .3, **b** *SSP* = *SSF* = .6, **c** *SSP* = *SSF* = .75. Encoding fixed at *a* = .3, and *cseq* = *csu* = .09. The solid diagonal line indicates chance performance. *SSP* Similarity of the innocent Suspect to the PERP, *SSF* Similarity of the Suspect to the Fillers, *PERP* perpetrator

Figure 3 shows the results of the theory space exploration of fair sequential lineups. The same 4 (lineup size: 1, 3, 6, and 12) × 3 (suspect/filler similarity (*SSP* = *SSF*): .3, .6, and .75) factorial was conducted, as for simultaneous lineups. Suspect position was randomly assigned for each simulation and performance was averaged across simulations. Panel A of Fig. 3 (*SSP* = *SSF* = .3) shows that the ROC truncates as lineup size increases from 1 to 12. Panels B (*SSP* = *SSF* = .6) and C (*SSP* = *SSF* = .75) do exhibit larger effects of adding fillers, but in the opposite direction predicted by filler siphoning theory. In fair sequential lineups, as more fillers are added, discriminability *decreases*. This is most likely due to the increased probability that a filler will be chosen before the model gets to the guilty suspects later in the lineup.

### Biased sequential lineups

**Fig. 4:**
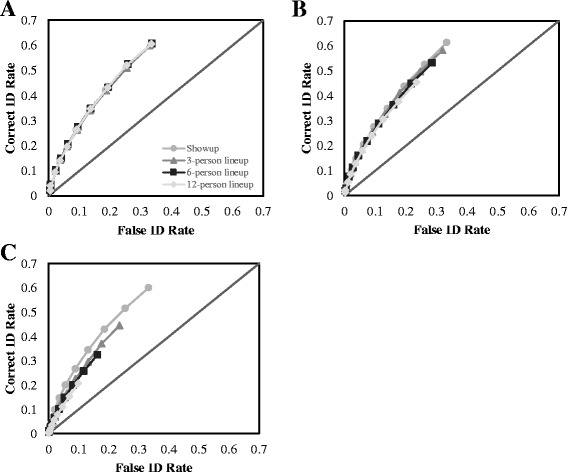
Receiver operating characteristic (ROC) curves comparing showups, 3-, 6-, and 12-person biased sequential lineups. The value of *SSP* = .75, making the innocent suspect a better match to the PERP than the fillers. **a** *SSF* = 0.0, **b** *SSF* = .3, **c** *SSF* = .6. Encoding constant at *a* = .3, and *cseq* = *csu* = .09. The solid diagonal line indicates chance performance. *SSP* Similarity of the innocent Suspect to the PERP, *SSF* Similarity of the Suspect to the Fillers, *PERP* perpetrator

Figure 4 shows the results of the 4 (lineup size: 1, 3, 6, and 12) × 3 (filler similarity (*SSF*): .0, .3, and .6) factorial for biased sequential lineups. Innocent suspect similarity (*SSP*) was held constant at .75 for these simulations. The results of the biased sequential lineup exploration are similar to the fair sequential lineup. Panel A (*SSF* = .0) shows no effect of number of fillers; panels B (*SSF* = .3) and C (*SSF* = .6) show that as fillers are added, discriminability decreases. In sequential lineups, fillers do not differentially protect the innocent suspect from identification.

In sum, by using a computational model that implements filler siphoning theory, we find evidence that siphoning can occur in simultaneous lineups and, if the fillers are competitive, produce lineup ROCs that are superior to showup ROCs (panel c in Figs. 1 and 2). But the advantage is small, which raises the question of whether the siphoning by fillers is sufficient to explain the magnitude of the lineup advantage seen in empirical data. In addition, we learned that filler siphoning theory does not generalize to sequential lineups. In what follows, we focus on the comparison of simultaneous lineups and showups because this is where we found some support for filler siphoning theory, and because this comparison remains central to Smith et al.’s (2017) arguments for their theory.

### Fitting empirical data

We fit WITNESS to the Wetmore et al. (2015) data, a set of data that has received a lot of attention in the academic literature. These data show a simultaneous lineup advantage over showups. We focused on the Wetmore et al. data from their Fig. 1, top panel, which collapsed over retention interval and lineup fairness (neither of which had significant effects on the partial areas under the respective ROC curves). If the presence of fillers is all that is needed to produce a simultaneous lineup advantage, then WITNESS should be able to approximate these data. To accomplish this, we can vary the number of fillers (6 vs. 1), and the values of the decision criteria (*csim* and *csu*). We must hold the other parameters constant across lineup and showup conditions because the ID procedure manipulation takes place after encoding, and the same innocent suspect is used in both conditions. If WITNESS can approximate these data given these constraints (values of *ssp* and *a* equal for lineups and showups), it suggests that filler siphoning theory is sufficient to explain the magnitude of the empirical performance differences between simultaneous lineups and showups.

**Fig. 5:**
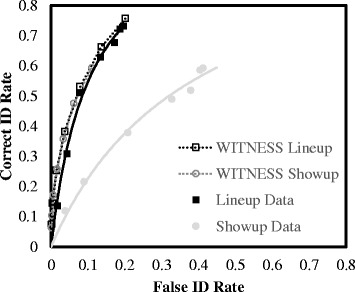
Empirical data from Wetmore et al. (2015) data and best fit to the data by the WITNESS model. Best-fitting parameters were: *a* = .31, *SSF* = .35, *SSP* = .50, *csim* = .07, *csu* = .095. *SSP* Similarity of the innocent Suspect to the PERP, *SSF* Similarity of the Suspect to the Fillers, *PERP* perpetrator

Table 1 reports the best fit obtained for the Wetmore et al. (2015) response proportion data, and Fig. 5 shows the empirical data and best-fitting WITNESS ROCs. Although WITNESS closely approximates the simultaneous lineup data, it mispredicts the extent of the showup performance deficit. As can be seen in Table 1 (values in bold), the model under-predicts the innocent suspect ID rate for showups. It would be possible to reverse this pattern and closely fit the showup data, but then the lineup data would be misfit. Irrespective of whether the showup or the lineup data serve as the “starting point,” the magnitude of the lineup advantage cannot be approximated. The failure of WITNESS to approximate these data is not a failure of the WITNESS model, but rather an indication that filler siphoning theory alone is insufficient to approximate the empirical data. Something more is needed.

**Table 1:** Wetmore et al. (2015) simultaneous lineup and showup data collapsed over retention interval and lineup fairness accompanied by WITNESS predictions

		Wetmore et al. (2015)		WITNESS	
		Target present	Target absent	Target present	Target absent
Showup	Suspect ID	.593	.*413*	.593	.*111*
	Rejection	.407	.587	.407	.889
Lineup	Suspect ID	.733	.196	.756	.202
	Filler ID	.087	.421	.110	.453
	Rejection	.180	.382	.134	.345

Target-absent lineup suspect ID rates are italicized to highlight the large degree of mis-fit in the WITNESS model in these cells

*ID* identification

### Embellishments to filler siphoning theory

A report by the National Academy of Sciences (National Research Council, 2014) raised an issue regarding the contribution of various sources of variability to the data used to construct ROC curves. An important characteristic of eyewitness research is that each participant provides only one decision, which means that between-participant variability may play a significant role. Thus far, the simulations have been treating each simulation/participant as if all simulations/participants utilize the same value of encoding or criterion. This is typical because researchers usually seek to fit average performance. However, it is possible that incorporating variability into our simulations might enhance the magnitude of difference between lineups and showups because variability may interact with ID procedure. Therefore, WITNESS was modified to enable exploration of variability and its relationship to performance. We explored both encoding and criterial variability (which have similar effects on performance), but focus on criterial variability because of the claims of Smith et al. (2017). First, however, we explore another suggestion by Smith et al. (Smith, Lindsay, Wells, & Myerson, 2016), who proposed that the criteria for target-present and target-absent lineups could differ.

### Criterion adjustment

In the version of the model we have presented up to this point, if two or more members of a lineup have match values that fall above criterion, the highest of these match values is chosen. However, Smith et al. (2016) proposed that the criteria for target-present and target-absent lineups could differ. To accomplish this, a mechanism must be added to the model such that it does not rely on the eyewitness knowing that a lineup is target-present or target-absent because that presupposes what the eyewitness is trying to determine. We propose that the criterion value is adjusted (made more conservative) whenever two or more match values fall above an initial criterion value, irrespective of whether the lineup was target-present or target-absent. We verified that this occurred more often in target-present lineups; on average, the guilty suspect is more likely to fall above criterion than the innocent suspect. This modification is similar, but distinct, from the One-Above-Criterion rule described in Clark et al. (2011), in which a decision is made if one and only one lineup member falls above criterion. According to our criterion adjustment modification, the best match is still chosen even if two or more lineup members fall above the adjusted criterion.^6^

**Fig. 6:**
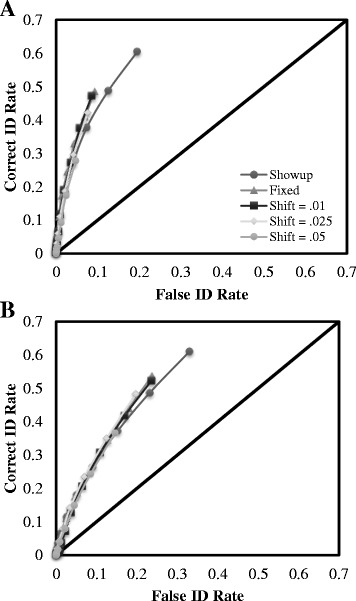
Criterion shift: receiver operating characteristic (ROC) curves comparing showups and 6-person lineups, with decision criterion fixed or allowed to shift + .01, + .025, and + .05, if two or more lineup members’ match values fell above *csim*. **a** Fair lineup, *SSF* = *SSP* = .6. **b** Biased lineup, *SSF* = .5, *SSP* = .75. *SSP* Similarity of the innocent Suspect to the PERP, *SSF* Similarity of the Suspect to the Fillers, *PERP* perpetrator

We implemented this modification in WITNESS and explored its effect on fair (*SSP* = *SSF* = .6) and biased (*SSP* = .75; *SSF* = .5) simultaneous lineups, compared to showups. For the lineups, we ran simulations where there was no criterion adjustment, a shift of + .01, + .025, or + .05. Figure 6 shows the results of these simulations; panel a shows the ROCs for a fair lineup and panel b shows the ROCs for a biased lineup. Adjusting the criterion to more conservative values did not affect discriminability beyond what we already had observed in Figs. 1 and 2 (in which there was no criterion adjustment). Criterion adjustment, as a modification to filler siphoning theory, failed to enhance the performance difference between simultaneous lineups and showups.

### Criterial variability

Each participant only provides one decision in an eyewitness task, and each participant likely approaches the task with a different decision criterion. One eyewitness, for example, may be troubled by the possibility of a wrongful conviction and adopts a conservative criterion; the next might be seeking vengeance and be eager to make a lineup selection. Smith et al. (2017) proposed that holding the decision criterion parameter constant when modeling eyewitness ROCs is inappropriate. However, it has been demonstrated within the basic memory literature that while criterial variability almost certainly exists (e.g., Benjamin, Diaz, & Wee, 2009; Wixted & Stretch, 2004), it likely does not produce major effects on the data (Kellen, Klauer, & Singmann, 2012). But these experiments were conducted to examine the impact of *within*-subject (and item) variability, whereas the eyewitness domain is concerned with *between*-subject variability, which could be more sizeable.

**Fig. 7:**
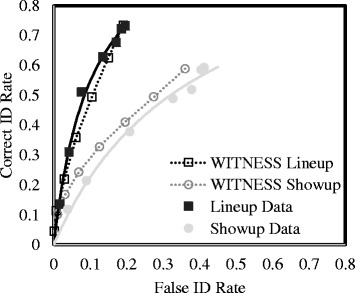
Empirical data from Wetmore et al. (2015) and best fit of the WITNESS model with criterial variability. Best fitting parameters were: *a* = .*38*, *SSF* = .45, *SSP* = .55, *csim* ~ *N*(μ = .07, = .80 * μ), *csu* ~ *N*(μ = .105, = .80 * μ). *SSP* Similarity of the innocent Suspect to the PERP, *SSF* Similarity of the Suspect to the Fillers, *PERP* perpetrator

To incorporate criterial variability, we simulated different eyewitnesses using different values of the decision criterion (Smith et al., 2017; see also McAdoo & Gronlund, 2016).^7^ The criterion value for each simulation was drawn from a normal distribution (μ = *csim*, or *csu*, σ = *y* * *csim*, or *csu*, where *y* is a scaling parameter, coupling the amount of variability to the value of *csim*, or, *csu*). The model fit improved quantitatively (*RMSD* = 0.012) with the inclusion of criterial variability. It also improved qualitatively because the magnitude of the lineup-showup difference is now comparable to empirical data (see Fig. 7).^8^

**Fig. 8:**
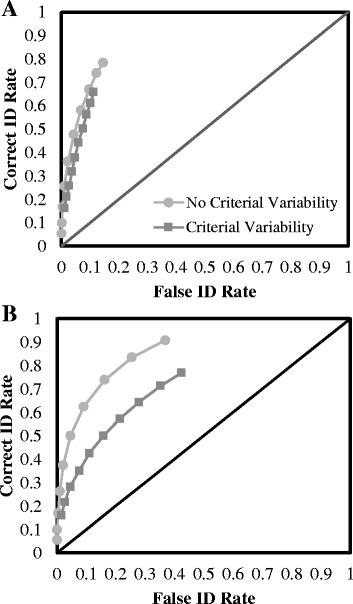
WITNESS-predicted receiver operating characteristics (ROCs) with and without criterial variability for: **a** simultaneous lineups, **b** and showups. Parameters for the no criterial variability condition were: *a* = .35, *SSF* = *SSP* = .5, *csim* = *csu* = .07. Parameter for the criterial variability condition were the same except that *csim* = *csu* ~ N(μ = .07, = .75 * μ). *SSP* Similarity of the innocent Suspect to the PERP, *SSF* Similarity of the Suspect to the Fillers, *PERP* perpetrator

Smith et al. (2017) argued that criterial variability was necessary for differential filler siphoning to operate, and that lineup performance would *improve* because choices would be increasingly siphoned away from innocent suspects. However, this was not case. When criterial variability is added to WITNESS, showups get *worse* (hits decrease, false alarms increase), but lineups essentially stay the same (hits and false alarms both decrease slightly). This is shown in Fig. 8, which depicts showup (panel b) and simultaneous lineup (panel a) performance with and without criterial variability. Criterial variability did not induce fillers to siphon more; instead, criterial variability adversely affected showup performance to a greater degree than lineup performance. In sum, WITNESS can now approximate the data, but not for the reason proposed by filler siphoning theory.

For the sake of completeness, we also examined the impact of criterial variability on sequential lineup performance. As with simultaneous lineups, adding increasing levels of criterial variability to a sequential lineup decision had little influence on discriminability, and the sequential ROCs were truncated. These results are a function of increased rates of early choices of fillers when the criterion is low, and lineup rejections when the criterion is high.

## Discussion

The primary goal of the present research was to determine if filler siphoning theory can explain why simultaneous lineups result in superior performance compared to showups. The WITNESS model was used to explore situations where siphoning should appear (by varying the number and quality of the fillers). Although we found that the choice of fillers in simultaneous lineups can provide some protection for an innocent suspect, by siphoning more choices from an innocent than a guilty suspect, this mechanism was insufficient to approximate empirical data. Like Smith et al. (2017), we found that incorporating criterial variability allowed WITNESS to approximate the magnitude of the lineup-showup difference seen in empirical data, but not because it induced more siphoning (enhancing lineup performance). Instead, criterial variability increased the magnitude of the lineup-showup performance difference by making showups worse (increasing false alarms and decreasing hits), and left simultaneous lineups relatively unaffected.

We also explored how filler siphoning theory impacts fair and biased sequential lineups. Our simulations revealed that fillers can have a detrimental effect on sequential lineup performance, thereby restricting the generalizability of filler siphoning theory. Notably, our simulations provide a theoretical rationale supporting the empirical conclusion (see Clark, Moreland, & Gronlund, 2014) that sequential lineups typically result in worse performance than simultaneous lineups. The presence of fillers can enhance simultaneous lineup performance, but the presence of fillers can harm sequential lineup performance.

There now exist three explanations for why simultaneous lineups induce superior performance to showups. We have focused on two, filler siphoning theory without criterial variability, and the greater adverse impact of criterial variability on showups than lineups. But we have yet to discuss the explanation Wetmore et al. (2015) relied on, the diagnostic feature hypothesis (Wixted & Mickes, 2014); the idea that simultaneous lineups result in better performance because they induce greater discriminability.

According to the diagnostic-feature hypothesis, fillers in simultaneous lineups afford access to better memory cues because an eyewitness can compare characteristics amongst the faces. An eyewitness is then able to identify those characteristics shared by all lineup members (and thus not helpful in distinguishing the perpetrator from the fillers), from those that are specific to the perpetrator. For example, if all lineup members are Caucasian with short, brown hair, race and hair color are irrelevant for identifying the perpetrator. By shifting attention away from these non-diagnostic characteristics, an eyewitness can focus on diagnostic characteristics unique to the perpetrator (e.g., a crooked nose). This comparison process is difficult in a showup because only one face is viewed, making it difficult for an eyewitness to determine which characteristics are diagnostic. The diagnostic feature hypothesis also can potentially explain why simultaneous lineups are superior to sequential lineups (e.g., Carlson & Carlson, 2014; Dobolyi & Dodson, 2013; Mickes et al., 2012), and why sequential suspect position effects are sometimes found (Carlson, Gronlund, & Clark, 2008; Gronlund, Carlson, Dailey, & Goodsell, 2009): An eyewitness viewing a sequential lineup has trouble determining which features are diagnostic until after viewing several lineup members. This explains why Gronlund et al. (2012) found that sequential lineups with the innocent or guilty suspect placed in position 2 resulted in no better performance than a showup, but sequential lineups with the suspect in position 5 were superior to a showup.

It is important to determine how fillers function to improve performance from simultaneous lineups. Policy recommendations—like conducting simultaneous lineups rather than showups—require theoretical support (see McQuiston-Surrett, Malpass, & Tredoux, 2006). The present work demonstrates that the explanation offered by filler siphoning theory is insufficient. That is, the mere presence of fillers, coupled with the notion that fillers siphon more choices from an innocent than a guilty suspect, is not an adequate explanation. Adding criterial variability does not make fillers siphon more. Instead, it adversely impacts showups more than simultaneous lineups because the addition of variability impacts a single match value from a showup to a greater extent than it does the best match of six from a lineup. Finally, fillers might increase discriminability, as Wetmore et al. (2015) claimed, but the diagnostic-feature model (Wixted & Mickes, 2014) has not yet been fit to data.

It was only by implementing filler siphoning theory in a formal model that its shortcomings were revealed. Likewise, it was only through implementing criterial variability in a formal model that we discovered that fillers may act to mitigate the impact of criterial variability—a new explanation that now warrants empirical testing. Hintzman (1991, p. 41) argued that one of the most important uses of formal models is to “clear up misconceptions and reveal underlying truths that are not obvious at first glance.” Therefore, it is crucial that the empirical exploration of these explanations be embedded within the context of formal models to accelerate the advance of the field (for similar calls see Clark, 2008; Clark & Gronlund, 2015; Gronlund et al., 2015; Wells, 2008).

## Additional file

Additional file 1: Attached Appendix contains overall identification rates as a function of lineup size and model parameters from the theory space exploration. (DOCX 24 kb)

## Footnotes

1 If a designated innocent suspect is not specified, then all IDs from the target-absent lineup are divided by the number of fillers to estimate a false ID rate.

2 It should be noted that previous research comparing lineups and showups relied on the *diagnosticity ratio* (correct IDs/false IDs), which ignored filler ID rates (e.g., Lindsay & Wells, 1985; Steblay, Dysart, & Wells, 2011).

3 We implemented WITNESS in R and the code used for our simulations is available upon request.

4 Clark et al. (2011) and Fife et al. (2014) have shown that in situations where the same fillers are used across target-present and target-absent lineups, the relative and absolute decision weights (*w*_a_ and *w*_r_, *w*_a_ + *w*_r_ = 1) have little impact on the model predictions (i.e., these decision weights trade-off with *csim*). In other words, the conclusions we reach in this article are unchanged if we had instead used a relative decision rule (take the difference between the best match and the next-best match and compare that to *csim*_r_) or a decision rule that weighed the absolute and relative contributions by *w*_a_ and *w*_r_, and compared that value to *csim*_ar_. That is, *a*, *SSP*, and *SSF* need not be modified to produce highly similar ROC curves across these different decision rules. Consequently, we chose to implement the simplest rule.

5 Although *csim*, *cseq*, and *csu* are decision criteria, we use different labels because they can take on different values. An eyewitness can see whether they are considering one, or more than one, face, and adjust a criterion accordingly.

6 This rule could not be implemented with a sequential lineup because WITNESS picks the first member it encounters that falls above criterion. There is no way to know if two members fell above criterion unless the entire lineup was viewed by the model, in which case the decision would be identical to a simultaneous lineup.

7 In other simulations, we varied the size of confidence “bins,” simulating noisy individual confidence thresholds. This modification did not change the pattern of the data.

8 We also fit the immediate fair innocent weak condition from Wetmore et al. (2015) and reached similar conclusions. Without criterial variability, we were unable to approximate the data. But after adding variability, we achieved a satisfactory qualitative match and good quantitative fit to the data (RMSD = .01; *a* = .34, *SSF* = .45, *SSP* = .50, *csim* ~ N(.06, .075*.06), *csu* ~ N(.08, .075*.08)).
