# Supplementary material for: The impact of fillers on lineup performance
Source: Cogn Res Princ Implic. 2017 Nov 22;2:48. doi: 10.1186/s41235-017-0084-1 (PMC5698388; doi:10.1186/s41235-017-0084-1)
Supplement: Supplementary file 1 — Attached Appendix contains overall identification rates as a function of lineup size and model parameters from the theory space exploration. (DOCX 24 kb) [file 41235_2017_84_MOESM1_ESM.docx]

Additional file 1:

Presented below are the overall identification rates as a function of lineup size and model parameters. The identification rates represent the cumulative performance from all participants (i.e., simulations in this case) including all levels of confidence.  As such, these numbers represent the right-most point on the ROC curve. We caution readers against interpreting these data as evidence for filler siphoning as presenting only these endpoints alone ignore the remainder of the ROC curves and can be misleading (see Gronlund, Mickes, Wixted, & Clark, 2015 for a similar argument concerning the sequential lineup advantage).  Furthermore, Rotello and Chen (2016) present arguments regarding drawing interpretations from a single data point, such as presented here.

*Predictions from WITNESS for the exploration of (1) fair simultaneous lineups, and (2) biased simultaneous lineups, (3) fair sequential lineups, (4) biased sequential lineups, and (5) showups and fair simultaneous lineups as criterial variability is added.*

| **1** |  | *SSP* = *SSF* = .3 | | | *SSP* = *SSF* = .6 | | | *SSP* = *SSF* = .75 | | |
| --- | --- | --- | --- | --- | --- | --- | --- | --- | --- | --- |
| Lineup Size | Target | Suspect | Filler | Reject | Suspect | Filler | Reject | Suspect | Filler | Reject |
| 1 | Present | 0.61 | NA | 0.39 | 0.61 | NA | 0.39 | 0.61 | NA | 0.39 |
|  | Absent | 0.04 | NA | 0.96 | 0.20 | NA | 0.80 | 0.33 | NA | 0.67 |
| 3 | Present | 0.60 | 0.02 | 0.38 | 0.55 | 0.09 | 0.36 | 0.51 | 0.15 | 0.35 |
|  | Absent | 0.04 | 0.08 | 0.88 | 0.14 | 0.27 | 0.59 | 0.18 | 0.37 | 0.45 |
| 6 | Present | 0.59 | 0.06 | 0.35 | 0.48 | 0.20 | 0.32 | 0.37 | 0.33 | 0.30 |
|  | Absent | 0.04 | 0.19 | 0.78 | 0.09 | 0.48 | 0.43 | 0.11 | 0.56 | 0.33 |
| 12 | Present | 0.56 | 0.11 | 0.32 | 0.38 | 0.36 | 0.26 | 0.22 | 0.54 | 0.24 |
|  | Absent | 0.03 | 0.33 | 0.64 | 0.06 | 0.64 | 0.30 | 0.07 | 0.70 | 0.24 |

Note: *a* = .3, *csim* = 0.09, *csu* = 0.09

| **2** |  | *SSF* = 0 | | | *SSF* = .3 | | | *SSF* = .6 | | |
| --- | --- | --- | --- | --- | --- | --- | --- | --- | --- | --- |
| Lineup Size | Target | Suspect | Filler | Reject | Suspect | Filler | Reject | Suspect | Filler | Reject |
| 1 | Present | 0.61 | NA | 0.39 | 0.61 | NA | 0.39 | 0.61 | NA | 0.39 |
|  | Absent | 0.35 | NA | 0.65 | 0.33 | NA | 0.67 | 0.34 | NA | 0.66 |
| 3 | Present | 0.60 | 0.00 | 0.39 | 0.60 | 0.02 | 0.38 | 0.57 | 0.08 | 0.36 |
|  | Absent | 0.34 | 0.00 | 0.65 | 0.32 | 0.05 | 0.63 | 0.26 | 0.21 | 0.53 |
| 6 | Present | 0.61 | 0.00 | 0.38 | 0.59 | 0.05 | 0.36 | 0.49 | 0.19 | 0.32 |
|  | Absent | 0.33 | 0.01 | 0.65 | 0.30 | 0.11 | 0.58 | 0.19 | 0.40 | 0.41 |
| 12 | Present | 0.61 | 0.02 | 0.37 | 0.56 | 0.10 | 0.33 | 0.38 | 0.36 | 0.26 |
|  | Absent | 0.36 | 0.03 | 0.64 | 0.27 | 0.22 | 0.50 | 0.12 | 0.59 | 0.29 |

Note: *a* = .3, *csim* = 0.09, *csu* = 0.09, *SSP =* .75

| **3** |  | *SSP* = *SSF* = .3 | | | *SSP* = *SSF* = .6 | | | *SSP* = *SSF* = .75 | | |
| --- | --- | --- | --- | --- | --- | --- | --- | --- | --- | --- |
| Lineup Size | Target | Suspect | Filler | Reject | Suspect | Filler | Reject | Suspect | Filler | Reject |
| 1 | Present | 0.61 | NA | 0.39 | 0.61 | NA | 0.39 | 0.61 | NA | 0.39 |
|  | Absent | 0.04 | NA | 0.96 | 0.20 | NA | 0.80 | 0.34 | NA | 0.66 |
| 3 | Present | 0.61 | 0.01 | 0.38 | 0.46 | 0.19 | 0.36 | 0.36 | 0.29 | 0.35 |
|  | Absent | 0.04 | 0.08 | 0.88 | 0.13 | 0.27 | 0.60 | 0.18 | 0.38 | 0.44 |
| 6 | Present | 0.53 | 0.16 | 0.37 | 0.33 | 0.47 | 0.32 | 0.23 | 0.56 | 0.30 |
|  | Absent | 0.03 | 0.37 | 0.80 | 0.09 | 0.32 | 0.44 | 0.12 | 0.56 | 0.33 |
| 12 | Present | 0.46 | 0.21 | 0.33 | 0.20 | 0.53 | 0.26 | 0.11 | 0.65 | 0.24 |
|  | Absent | 0.03 | 0.33 | 0.65 | 0.06 | 0.64 | 0.30 | 0.07 | 0.70 | 0.24 |

Note: *a* = .3, *csim* and *csu* = 0.09

| **4** |  | *SSF* = .3 | | | *SSF* = .6 | | | *SSF* = .75 | | |
| --- | --- | --- | --- | --- | --- | --- | --- | --- | --- | --- |
| Lineup Size | Target | Suspect | Filler | Reject | Suspect | Filler | Reject | Suspect | Filler | Reject |
| 1 | Present | 0.60 | NA | 0.40 | 0.61 | NA | 0.39 | 0.60 | NA | 0.40 |
|  | Absent | 0.34 | NA | 0.66 | 0.33 | NA | 0.67 | 0.33 | NA | 0.67 |
| 3 | Present | 0.60 | 0.00 | 0.40 | 0.58 | 0.04 | 0.37 | 0.44 | 0.19 | 0.37 |
|  | Absent | 0.33 | 0.00 | 0.67 | 0.32 | 0.06 | 0.63 | 0.24 | 0.24 | 0.53 |
| 6 | Present | 0.61 | 0.01 | 0.38 | 0.53 | 0.10 | 0.36 | 0.32 | 0.36 | 0.32 |
|  | Absent | 0.34 | 0.01 | 0.65 | 0.29 | 0.14 | 0.58 | 0.16 | 0.43 | 0.41 |
| 12 | Present | 0.61 | 0.03 | 0.37 | 0.45 | 0.22 | 0.33 | 0.21 | 0.53 | 0.26 |
|  | Absent | 0.34 | 0.03 | 0.63 | 0.23 | 0.26 | 0.50 | 0.10 | 0.62 | 0.28 |

Note: *a* = .3, *csim* and *csu* = .09, and *SSP* = .75

| **5** |  | *Variability Multiplier = .00* | | | *Variability Multiplier = .25* | | | *Variability Multiplier = .75* | | |
| --- | --- | --- | --- | --- | --- | --- | --- | --- | --- | --- |
|  | Target | Suspect | Filler | Reject | Suspect | Filler | Reject | Suspect | Filler | Reject |
| Showup | Present | 0.91 | NA | 0.09 | 0.88 | NA | 0.12 | 0.77 | NA | 0.23 |
|  | Absent | 0.37 | NA | 0.63 | 0.38 | NA | 0.62 | 0.42 | NA | 0.58 |
| Lineup | Present | 0.78 | 0.15 | 0.06 | 0.76 | 0.15 | 0.09 | 0.66 | 0.13 | 0.21 |
|  | Absent | 0.15 | 0.69 | 0.16 | 0.14 | 0.65 | 0.20 | 0.11 | 0.55 | 0.33 |

Note: *a* = .35, *SSF* = *SSP* = .5, *csim* = 0.07, *csu* = 0.07
